# Graves’ Disease in a Patient With Human Immunodeficiency Virus Infection as an Immune Reconstitution Inflammatory Syndrome

**DOI:** 10.7759/cureus.15377

**Published:** 2021-06-01

**Authors:** Sarah Ayad, Kirolos Gergis, Noreen Mirza, Mohammad Nabil Rayad, Julius Salamera

**Affiliations:** 1 Internal Medicine, Rutgers-New Jersey Medical School/Trinitas Regional Medical Center, Elizabeth, USA; 2 Internal Medicine, McLaren Flint Hospital, Flint, USA; 3 Internal Medicine, Trinitas Regional Medical Center, Elizabeth, USA

**Keywords:** graves’ disease, hyperthyroidism, immune reconstitution inflammatory syndrome, haart, hiv

## Abstract

The use of highly active antiretroviral therapy (HAART) in the management and treatment of human immunodeficiency virus type 1 (HIV-1) has dramatically changed the course of the disease and improved overall survival. HAART results in significant decrease in viral load and enhancement of CD4 cells and gradual restoration of the immune system. However, a subset of patients may experience a paradoxical worsening after the initiation of HAART due to a heightened and dysregulated immune response. This phenomenon is termed immune reconstitution inflammatory syndrome (IRIS). The manifestation of Graves’ disease (GD) after the introduction of HAART has been identified as IRIS manifestation in some patients. Thus, this occurrence should be suspected and further investigated in patients with HIV on antiretroviral therapy (ART) who present with symptoms consistent of hyperthyroidism to avoid overt hyperthyroidism. We report a case of IRIS associated Graves’ disease. Our case adds to the very limited literature about this phenomenon.

## Introduction

Human immunodeficiency virus (HIV), which causes acquired immunodeficiency syndrome (AIDS), is a major public health issue that has led to major cultural, economic, and health suffering that affected almost every population worldwide. As of 2016, approximately 36.7 million individuals globally live with HIV/AIDS [1]. In United States, there are roughly 1.1 million people living with HIV (PLHIV) [2]. The development and increased access to highly active antiretroviral therapy (HAART) in the management of HIV histrionically changed the course of the disease and improved overall survival and reduction in morbidity and mortality [3,4]. In the HAART era, there has been about 28% decrease in deaths related to HIV from 2006 to 2012 [3]. 

HIV is associated with a decrease in CD4+ memory cell count and thymic dysfunction, and an increase in the number of activated T cells in the peripheral blood [5]. The introduction of HAART results in decreased morbidity and mortality via restoration of the previously compromised immune system [6]. However, a subset of patients may experience a paradoxical worsening after the initiation of HAART, a phenomenon called Immune reconstitution inflammatory syndrome (IRIS) [6,7,8]^.^ IRIS has been defined as a dysfunction of restored immune responses after HAART initiation, typically in patients with the lowest CD4 count [9]. In this syndrome, a heightened inflammatory reaction occurs in response to either an infectious or non-infectious antigen that was previously obscure to the immune system [7,8]. IRIS is associated with fungal infections, herpes viruses and is commonly seen with mycobacterial and cryptococcal infections [8,9].^ ^ Furthermore, multiple autoimmune conditions, either novel disorders or acute flare ups of existing autoimmune conditions, were identified in patients receiving HAART [7,8]. In a limited number of case reports, Graves' disease and sarcoidosis were identified as possible autoimmune manifestations of IRIS [9].^ ^ Approximately 3% of women and 0.2% of men developed Graves’ disease 8-33 months after starting HAART [10]. Here, we report a case of IRIS-associated Graves’ disease.

## Case presentation

A 43-year-old African American female with HIV infection for seven years has a history of various opportunistic infections including *Pneumocystis jirovecii *pneumonia, crytococcemia, *Cytomegalovirus *retinitis, and disseminated *Mycobacterium avium *intracellulare. Her compliance to highly active antiretroviral therapy** **has been variable with ongoing viremia over the last five years, and consistently low CD4 count of less than 100. She presented to the emergency room with a four-day history of nausea, vomiting, diarrhea, cough, and subjective fevers associated with tenderness on the left side of her neck. There is no history of thyroid dysfunction, neck irradiation, family history of thyroid disease, recent travel, or recent sick contacts. 

**Figure 1:**
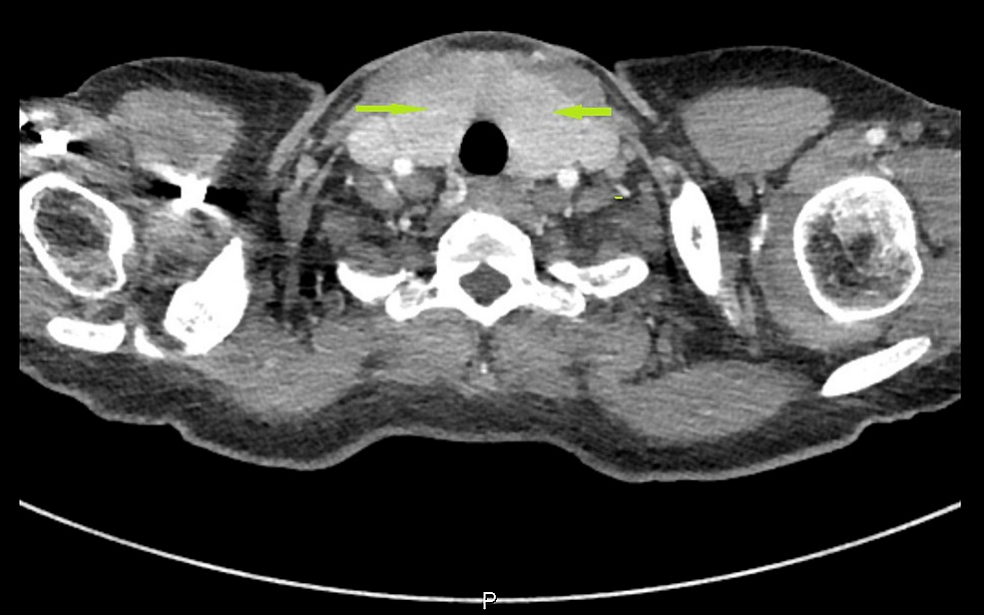
CT imaging showing markedly enlarged heterogeneous thyroid gland with prominent cervical lymphadenopathy (arrows)

**Figure 2:**
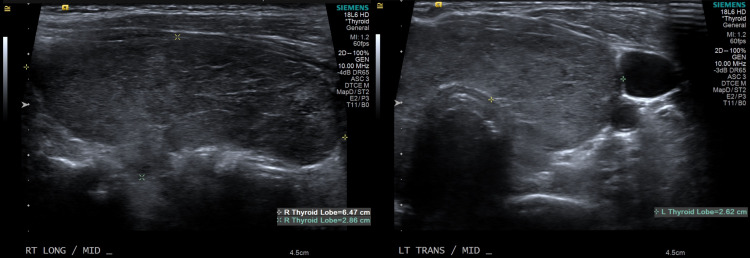
Ultrasound revealed enlarged heterogeneous thyroid gland

**Figure 3:**
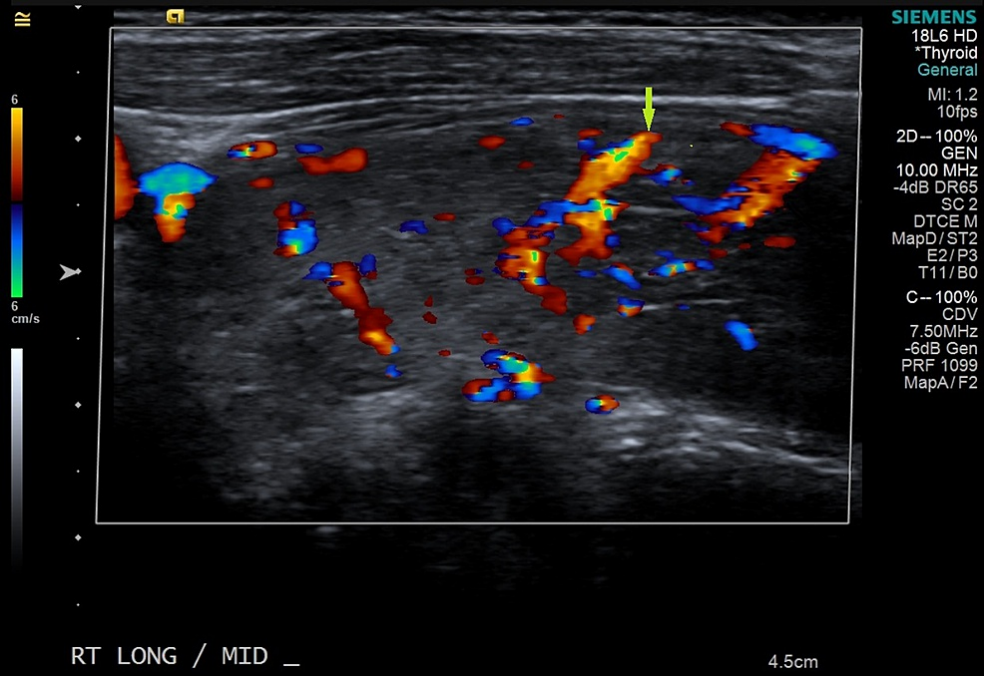
Ultrasound revealed enlarged heterogeneous thyroid gland with increased vascularity

On examination, she was hypertensive, tachycardic, and later developed fever as high as 101.6 degrees Fahrenheit. The neck was supple with a prominent thyroid gland, more on the left, associated with tenderness on palpation. Thyroid function tests revealed elevated free T4 of 5.5 [0.61-1.12 ng/dl] and T3 of 7.6 [0.87-1.78 ng/ml] with thyroid-stimulating hormone (TSH) level of 0.01 [0.34-5.6 ulU/ML]. The electrocardiogram disclosed sinus tachycardia with ventricular rate of 135 and non-specific T-wave abnormality with no ischemic changes. CT pulmonary angiogram ruled out central pulmonary embolic disease, but incidentally noted a markedly enlarged heterogeneous thyroid gland with prominent cervical lymphadenopathy (Figure 1). Thyroid ultrasound revealed enlarged heterogeneous thyroid gland with increased vascularity (Figures 2, 3). Thyroid-stimulating immunoglobulin turned out to be abnormal at 324% of the reference control. The patient was started on methimazole 30 mg daily along with propranolol 40 mg four times a day, with improvement of her clinical symptomatology. At the time, repeat HIV parameters revealed improvement in virologic control while on bictegravir/emtricitabine/tenofovir alafenamide and a CD4 count of 330.

## Discussion

HIV targets CD4+ T-cells which in turn results in immunosuppression and an increase in the susceptibility to opportunistic pathogens. ART is used to treat HIV and it does so by dramatically increasing the number of CD4+ T-cells restoring immune function [9]. Since 1990, when ART was developed, the rates of morbidity and mortality secondary to HIV have been largely reduced [11]. Although ART has a pivotal role in combating HIV infection rates, it may cause a paradoxical deterioration in the health of those patients being treated [9]. In particular, patients with a very low nadir CD4+ T-cell counts are more likely to be affected by this paradox known as IRIS [11]. Although the exact mechanism of this phenomenon is not very well understood, it is thought to be related to immune function reactivation resulting in increased ability to fight infectious and noninfectious antigens [11]. 

IRIS tends to occur within a few weeks and up to several months after the initiation of HAART. It has been linked to the reactivation of infectious pathogens such as *Mycobacterium avium* complex, *Mycobacterium tuberculosis*, *Cytomegalovirus*, and *Cryptococcus neoformans* [6]. Autoimmunity may occur as a rare consequence of HAART initiation. In particular, patients with HIV who initiated HAART therapy may develop Graves’ disease (GD) [6]. GD as a consequence of IRIS after ART initiation is similar to the conventional GD type; thus, they are both treated similarly [6]. Here, we report on the occurrence of GD, an autoimmune disorder, which occurred very late into the treatment course. 

This phenomenon was first described by Gilquin et al. in 1998 [12]. The number of IRIS-induced GD cases that have occurred so far has been low. In our patient, a diagnosis of GD was made after the initiation of HAART totaled 16 months. The median CD4 T-cell nadir value was 23 cells/μL and an increase of CD4 T-cell count of 330 cells/μL was seen before the patient could be diagnosed with hyperthyroidism. Like other cases seen in the literature, in our patient, there was no history of GD or any other autoimmune diseases. 

The pathogenesis of IRIS induced GD is thought to occur during the second phase of HAART initiation [5]. During the first phase of therapy - first few months - HIV replication is inhibited and memory T-cells [5] are released from the inflamed lymphoid tissue [6]. This causes a rapid increase in the number of CD4 T-cell numbers. During the second phase of therapy which happens months to years later, the thymus releases naïve CD4+ T cells at a slower rate [5]. Initiation of HAART causes an intense regeneration of the thymus and the naïve CD4 T-cells are unable to suppress autoreactive cells resulting in the development of thyroid peroxidase (TPO) antibodies and TSH receptor antibodies [6]. Another mechanism that has been related to HAART-induced GD is thought to be due to molecular mimicry between the TSH receptor (TSH-R) and immunogenic proteins from HIV [11]. There has also been a relation to certain major histocompatibility complex (MHC) genes and polymorphisms in cytokines [6].

In 1998, Gilquin et al. described the first three cases of GD as a phenomenon of HAART immune reconstitution developing after 16-22 months of therapy [12]. In 2005, Chen et al., described a case where 17 patients (65% were Black African or Black Caribbean ethnicity, and 85% were females) were diagnosed with HAART-induced GD with a median time of onset after 17 months of therapy [5]. In 2020, Nallu et al., described a case of a patient with no personal history but a family history of GD that developed GD 24 months after initiation of HAART [11]. In all of the studies described above, and as seen in our case, there was a strong relationship between the rise of CD4+ T cell count, drop in HIV viral load, and the onset of GD. 

Management of GD associated with HAART is the same treatment as for conventional GD. Rasul et al, described four patients who developed Graves’ Disease via IRIS and were managed successfully with antithyroid drugs including methimazole and propylthiouracil (PTU) and had complete resolution of symptoms [6]. One of the patients required I-131 radioablation in addition to methimazole [6]. Our patient was started on methimazole 30 mg daily and propranolol 40 mg qid, which is also used for conventional GD. After initiation of therapy, there was a significant improvement in clinical symptoms. Overall, it is important to monitor HIV positive patients on anti-retroviral therapy for the development of any manifestations of IRIS-induced GD. It is important to be attentive since these cases may occur even years after therapy. This patient did not have overt clinical signs of hyperthyroidism; thus, clinicians may consider monitoring TSH levels periodically. 

## Conclusions

The introduction of HAART in the management of HIV has led to dramatic improvement of survival and decreased morbidity and mortality. However, in some patients the introduction of HARRT can lead to worsening of the clinical course in a phenomenon called IRIS. Due to the wide clinical presentation and the growing variety of manifestations, physicians ought to be vigilant and cautious when initiating ART to prevent detrimental side effects. Graves’ disease as manifestation of IRIS should be assumed in patients on HAART and exhibit symptoms consistent with hyperthyroidism and measuring a TSH level is suitable in these cases.
